# Über Puerperale Psychosen

**Published:** 1897-12

**Authors:** 


**Über Puerperale Psychosen.** Von Dr. Oswald Knauer. Pp. 54. Berlin: S. Karger. 1897.

We welcome this short treatise on the causation of mental affections common to the puerperal state, as a useful contribution to an important subject. The author gives a minute analysis of a large number of cases under his care, and classifies them under the heads of: (1) those arising from febrile infectious diseases; (2) those types arising without fever; (3) and those dependent on uræmia and eclampsia. The reader will find much valuable material for the study of these diseases collected by a man of large experience, and well arranged for reference.

